# Effects of aesthetic suturing on wound healing and scar formation in patients with traffic accident–related injuries

**DOI:** 10.3389/fsurg.2026.1804506

**Published:** 2026-04-13

**Authors:** Jun Ren, Jing Diao, Ya-Qin Zhang, Chen Cai, Zi-Liang Gong, Bi-Bo Tang

**Affiliations:** 1 Department of Emergency Medicine, The Third People's Hospital of Hubei Province, Wuhan, Hubei, China; 2 Department of Neurology, The Third People's Hospital of Hubei Province, Wuhan, Hubei, China; 3 Department of Orthopedics, The Third People's Hospital of Hubei Province, Wuhan, Hubei, China

**Keywords:** aesthetic suturing, hypertrophic scar, subgroup analysis, surgical site infection, traffic accident injuries, traumatic laceration, Vancouver scar scale, wound healing

## Abstract

**Introduction:**

Traffic accident–related lacerations frequently require urgent primary closure and may be complicated by irregular wound margins, heterogeneous contamination, and variable mechanical tension, all of which can influence scar maturation. This study evaluated the association between aesthetic suturing and clinical wound healing as well as scar-related outcomes in patients with traffic accident–related injuries.

**Methods:**

In this retrospective observational study, 117 consecutive patients treated between March 2023 and September 2024 were categorized according to the closure approach documented at index repair (aesthetic suturing, *n* = 58; conventional suturing, *n* = 59). Data were extracted from electronic medical records using a standardized case report form. Outcomes included time to complete healing, primary healing, wound-related complications, healthcare utilization after discharge, and scar/aesthetic outcomes assessed by the Vancouver Scar Scale (VSS), symptom scores, and patient satisfaction.

**Results:**

Time to complete healing and primary healing were comparable between groups, and no significant differences were observed in surgical site infection, dehiscence, hematoma/seroma, marginal ischemia/necrosis, or secondary procedures. Revisit/readmission was less frequent after aesthetic suturing. Scar outcomes favored aesthetic suturing, with substantially lower VSS total and domain scores, lower scar pain, and higher patient satisfaction; hypertrophic scar and contracture rates did not differ significantly. Subgroup analyses showed consistent VSS benefit without significant interaction across prespecified strata.

**Discussion:**

Aesthetic suturing was associated with improved scar quality and patient-reported aesthetic outcomes without an apparent increase in short-term wound complications.

## 1 Introduction

Traumatic lacerations related to road traffic accidents represent a frequent indication for acute wound closure and are often accompanied by irregular wound edges, variable contamination, and heterogeneous mechanical loading across anatomic regions (1). Beyond restoring barrier integrity, contemporary trauma care increasingly emphasizes patient-centered outcomes, including scar appearance, scar-related symptoms, and return to normal social functioning, particularly when wounds involve cosmetically sensitive regions or high-tension/joint-adjacent sites where hypertrophic scarring and functional limitation may occur (2, 3). Recent reviews of hypertrophic scarring and keloids summarize that pathological scar formation reflects a complex interaction among prolonged inflammation, aberrant angiogenesis, fibroblast activation, and extracellular matrix remodeling, with mechanical forces acting as a persistent amplifier of fibrotic signaling during the remodeling phase (4, 5). While early wound complications remain core safety outcomes in traumatic wound management, multiple contemporary studies and reviews in laceration repair indicate that different closure strategies may yield broadly similar short-term complication rates yet diverge in long-term cosmetic endpoints (6, 7). For example, comparative studies in facial lacerations have evaluated tissue adhesives vs. sutures, focusing on wound complications and cosmetic performance under real-world emergency care conditions (6). In parallel, randomized evidence in adults has strengthened the feasibility of technique selection based on downstream appearance without compromising basic safety endpoints (8). These lines of evidence support the clinical premise that closure method and suture execution may influence scar maturation even when early epithelialization is not markedly altered (9).

Aesthetic suturing is conceptually positioned to optimize scar outcomes through precise dermal alignment, tension redistribution, and meticulous epidermal approximation, thereby potentially mitigating microischemia, suture-track inflammation, and maladaptive remodeling (10). The role of mechanical tension in scar modulation is supported by emerging evidence indicating that mechanical forces influence inflammation and collagen remodeling during cutaneous repair (11). However, trauma populations differ from elective surgical cohorts because injury mechanism, tissue crush, contamination, and presentation timing can vary substantially, which may modify the net benefit of aesthetic techniques and complicate translation of elective-scar paradigms to acute trauma care (10). Robust evaluation of scar formation also depends on the availability of validated scar assessment instruments that capture both clinician- and patient-relevant dimensions (12). Recent methodological work has advanced the Patient and Observer Scar Assessment Scale (POSAS) toward updated versions and cross-cultural validation, supporting its use across scar etiologies, including trauma (8, 13). In addition, updated reviews of wound-closure technologies and postoperative scar management have highlighted the expanding toolkit for optimizing cutaneous outcomes and the need for context-specific evidence that integrates procedural technique with pragmatic follow-up and patient education (14).

Against this background, systematic clinical assessment of aesthetic suturing in traffic accident–related injuries may clarify its real-world association with wound healing trajectories, complication profiles, scar quality, symptom burden, and patient satisfaction across clinically relevant subgroups.

## 2 Methods

### 2.1 Study design

This retrospective observational study consecutively enrolled patients with traffic accident–related injuries who underwent wound repair at our institution between March 2023 and September 2024. Eligible participants met the following inclusion criteria: (1) a documented history of traffic accident–related traumatic injury requiring primary wound closure; (2) wounds amenable to direct suturing (without the need for flap reconstruction or skin grafting at the index procedure); (3) complete perioperative clinical records, including wound characteristics and procedural details; and (4) availability of follow-up assessments sufficient to evaluate wound healing outcomes and scar formation. Exclusion criteria were: (1) extensive tissue loss, crush injury, or compartment syndrome requiring staged reconstruction; (2) severe wound contamination or established infection at presentation; (3) concomitant burns or chemical injuries involving the wound area; (4) immunodeficiency or current systemic immunosuppressive therapy; (5) malignant disease or other conditions precluding standardized follow-up; and (6) incomplete data on key outcomes. Patients were categorized according to the suturing approach used at the index repair (aesthetic suturing vs. conventional suturing) based on operative documentation. The study was reviewed and approved by the institutional ethics committee. All procedures were conducted in accordance with relevant guidelines and the Declaration of Helsinki. Informed consent was obtained from all participants. Data were anonymized prior to analysis to ensure confidentiality and protect participant privacy.

### 2.2 Data collection

Data were retrospectively extracted from the institutional electronic medical record (EMR) system for all eligible patients with traffic accident–related injuries who underwent primary wound closure during the study period. A standardized case report form (CRF) was used to ensure uniform variable definitions and coding. Two trained investigators independently abstracted data from emergency department records, procedure/operative notes, nursing documentation, medication administration records, laboratory and microbiology reports, discharge summaries, outpatient follow-up notes, and readmission records. Discrepancies were resolved by consensus, with senior clinician adjudication when required. Baseline data included demographics (age, sex, body mass index), smoking status, and comorbidities (e.g., diabetes mellitus and hypertension) recorded at presentation. Injury- and wound-related variables were obtained from initial assessment and procedure documentation, including time from injury to presentation/closure (hours), wound location (face, scalp, extremity, trunk), wound length (cm) and estimated area (cm^2^), wound depth category, high-tension/joint-region involvement, wound edge characteristics (regular vs. irregular), and contamination grade (clean, contaminated, severely contaminated) based on prespecified criteria aligned with clinical documentation.

Treatment-related variables included anesthesia type, irrigation/debridement approach, prophylactic antibiotic use, closure strategy (single-layer vs. layered), and suture material when recorded. Wound preparation/debridement was classified into three predefined categories: irrigation only, standard debridement, and extensive debridement. Irrigation only referred to copious saline irrigation without excision of nonviable tissue. Standard debridement referred to routine wound bed preparation with saline irrigation followed, when clinically indicated, by limited sharp debridement of clearly devitalized tissue, foreign material, and gross contaminants. Extensive debridement referred to more thorough sharp/surgical excision and repeated cleansing for wounds with broader zones of contusion, embedded debris, or more complex contamination. The exposure (aesthetic vs. conventional suturing) was classified according to the procedural description and standardized criteria; ambiguous cases were reviewed by a senior clinician. Outcome data were captured from inpatient and follow-up records, including time to complete healing, primary healing status, wound-related complications (surgical site infection, dehiscence, hematoma/seroma, marginal ischemia/necrosis), secondary interventions (repeat debridement and/or re-suturing), and unscheduled revisit/readmission. Scar and aesthetic outcomes were extracted from follow-up assessments, including Vancouver Scar Scale (VSS) total and domain scores, hypertrophic scar and contracture (when applicable), scar symptoms (itch/pain scores using NRS/VAS if documented), and patient-reported satisfaction (Likert scale when available). Data underwent range and logic checks, and missing fields were documented for each variable prior to analysis.

### 2.3 Aesthetic suturing definition and outcome assessment

In this study, aesthetic suturing referred to closure techniques intended to optimize subsequent scar quality, typically emphasizing careful wound-edge alignment, atraumatic tissue handling, minimization of closure tension, and layered approximation when required by wound depth and configuration. Conventional suturing referred to routine traumatic wound closure primarily aimed at wound approximation and protection, without explicit documentation of cosmetic-oriented closure principles. Classification was based on operative documentation, and cases with unclear descriptions were reviewed and adjudicated by a senior clinician. Aesthetic suturing generally involved layered closure when wound depth, dead space, or tension distribution indicated the need for deeper support. Buried dermal sutures were used when appropriate to reduce superficial tension and assist wound-edge approximation. Skin closure emphasized precise edge apposition with slight eversion when feasible, avoidance of excessive strangulation, and relatively fine, closely spaced sutures adapted to wound location, length, and local tension. The choice between interrupted and continuous closure was determined by wound characteristics and operator judgment; interrupted skin sutures were more commonly used for irregular traumatic lacerations, whereas continuous closure could be applied selectively in suitable low-tension wounds with well-aligned edges.

Scar-related outcomes were assessed from follow-up records considered sufficient for wound-healing and scar evaluation. The VSS was used to evaluate scar quality, including vascularity, pigmentation, pliability, and height/thickness, with total and domain scores extracted from documented follow-up assessments. Scar symptoms (itch and pain), hypertrophic scar, contracture, and patient-reported satisfaction were also recorded when available. Scar assessment was based on routine in-person clinical follow-up conducted as part of routine clinical care. To improve data reliability, two trained investigators independently abstracted scar-related data using a standardized case report form, and discrepancies or ambiguous entries were resolved by consensus with senior clinician adjudication. The investigators responsible for data abstraction were trained in predefined variable definitions and medical-record review procedures; outcome classification relied on documented clinical assessments in the follow-up record.

### 2.4 Statistical analysis

All analyses were conducted using IBM SPSS Statistics, version 26.0 (IBM Corp., Armonk, NY, USA). Data distribution was evaluated using histograms and the Shapiro–Wilk test. Continuous variables are presented as mean ± standard deviation when approximately normally distributed and as median [interquartile range] when non-normally distributed. Between-group comparisons for continuous variables were performed using the independent-samples t test (normal distribution) or the Mann–Whitney U test (non-normal distribution/ordinal data). Categorical variables are summarized as *n* (%) and compared using the Pearson *χ*^2^ test; Fisher's exact test was applied when expected cell counts were <5. Subgroup analyses were performed using stratified comparisons with the same statistical tests. All tests were two-sided, and *P* < 0.05 was considered statistically significant.

## 3 Results

### 3.1 Baseline demographic and injury characteristics

**Figure 1:**
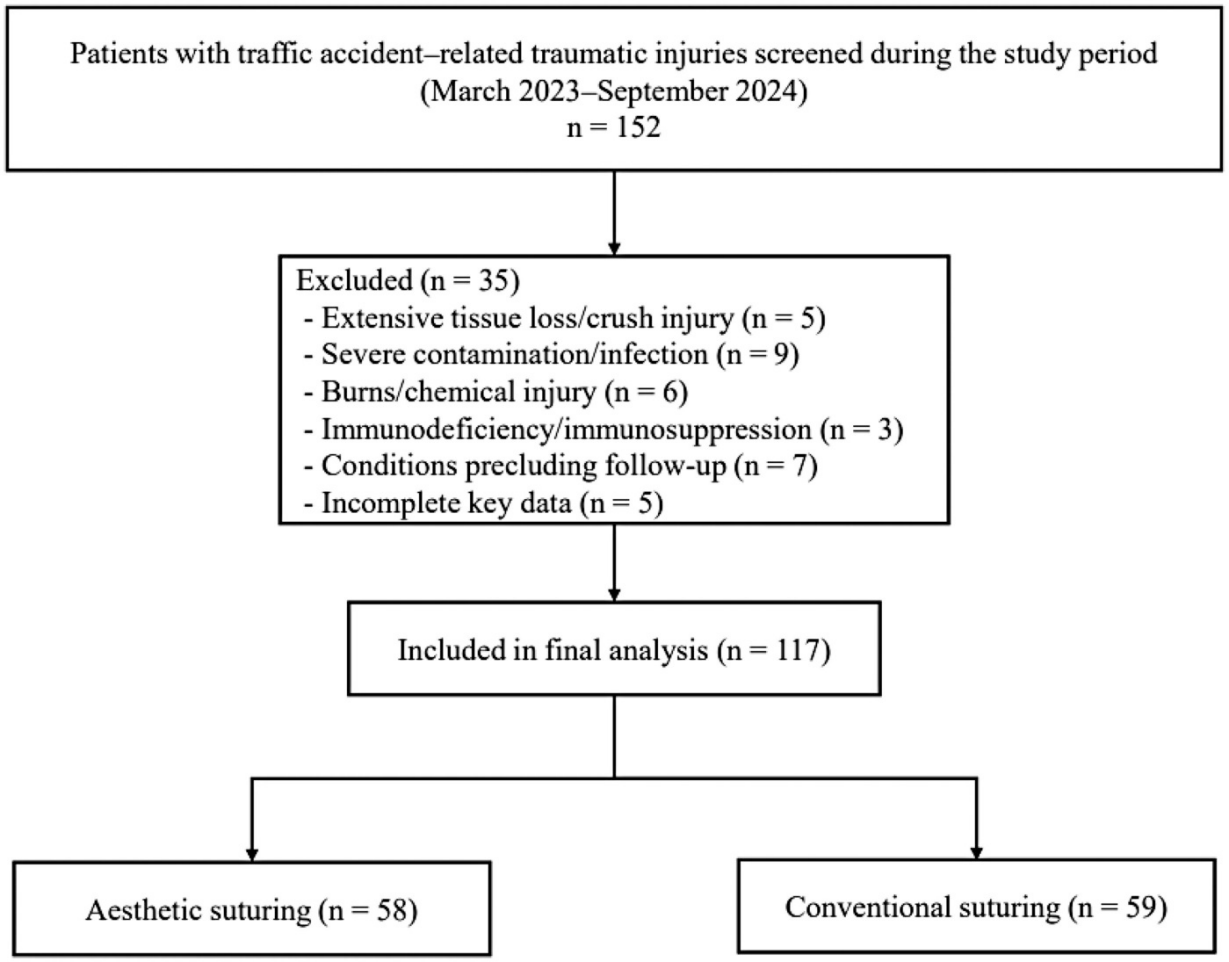
Flowchart of patient selection.

A total of 117 patients with traffic accident–related injuries were included, comprising 58 patients in the aesthetic suturing group and 59 patients in the conventional suturing group (Figure 1). Baseline demographic characteristics were broadly comparable between groups. The mean age was 36.52 ± 10.83 years in the aesthetic suturing group and 37.22 ± 11.51 years in the conventional suturing group (t = −0.34, *P* = 0.733). The proportion of male patients was similar (62.1% vs. 61.0%; *χ*^2^ = 0.00, *P* = 1.000), and body mass index did not differ significantly between groups (24.38 ± 3.32 vs. 24.20 ± 2.83 kg/m^2^; t = 0.32, *P* = 0.749). Smoking status distribution (never/former/current) was also comparable (*χ*^2^ = 0.29, *P* = 0.867). With respect to comorbidities, the prevalence of diabetes mellitus (17.2% vs. 10.2%; *χ*^2^ = 0.71, *P* = 0.399) and hypertension (22.4% vs. 20.3%; *χ*^2^ = 0.00, *P* = 0.962) did not differ between groups. Injury- and wound-related characteristics demonstrated no significant differences for most variables. Median injury-to-presentation time was 3.74 [2.65, 5.00] hours in the aesthetic suturing group and 4.19 [3.15, 6.08] hours in the conventional suturing group (U = 1,458, *P* = 0.169). Wound location distribution (face/scalp/extremity/trunk) was similar across groups (*χ*^2^ = 5.01, *P* = 0.171). However, the conventional suturing group presented with a greater wound length (6.20 ± 2.45 vs. 5.19 ± 2.41 cm; t = −2.25, *P* = 0.027) and larger wound area (7.83 ± 3.91 vs. 5.91 ± 3.30 cm^2^; t = −2.86, *P* = 0.005). Wound depth category, involvement of high-tension areas, edge regularity, and contamination grade were comparable (all *P* > 0.05). Management-related baseline characteristics were balanced between groups. The distributions of anesthesia type and debridement approach did not differ significantly (*P* = 0.596 and *P* = 0.324, respectively). Use of prophylactic antibiotics (69.0% vs. 64.4%; *χ*^2^ = 0.11, *P* = 0.744), layered closure (51.7% vs. 61.0%; *χ*^2^ = 0.68, *P* = 0.408), and suture material selection (*χ*^2^ = 1.78, *P* = 0.410) were also similar between groups (Table 1).

**Table 1:** Baseline demographic, injury, and treatment-related characteristics.

Variable	Aesthetic suturing (*n* = 58)	Conventional suturing (*n* = 59)	Test statistic	*P* value
Age, years	36.52 ± 10.83	37.22 ± 11.51	t = −0.34	0.733
Male sex, *n* (%)	36 (62.1)	36 (61.0)	*χ*^2^ = 0.00	1.000
BMI, kg/m^2^	24.38 ± 3.32	24.20 ± 2.83	t = 0.32	0.749
Smoking status (Never/Former/Current), *n* (%)	34 (58.6)/13 (22.4)/11 (19.0)	36 (61.0)/14 (23.7)/9 (15.3)	χ^2^ = 0.29	0.867
Diabetes mellitus (DM), *n* (%)	10 (17.2)	6 (10.2)	χ^2^ = 0.71	0.399
Hypertension, *n* (%)	13 (22.4)	12 (20.3)	χ^2^ = 0.00	0.962
Injury-to-presentation time, hours	3.74 [2.65, 5.00]	4.19 [3.15, 6.08]	U = 1,458	0.169
Wound location (Face/Scalp/Extremity/Trunk), *n* (%)	26 (44.8)/8 (13.8)/16 (27.6)/8 (13.8)	18 (30.5)/5 (8.5)/27 (45.8)/9 (15.3)	χ^2^ = 5.01	0.171
Wound length, cm	5.19 ± 2.416543	6.20 ± 2.45	t = −2.25	0.027
Wound area, cm^2^	5.91 ± 3.30	7.83 ± 3.91	t = −2.86	0.005
Wound depth (Superficial/Partial-thickness/Full-thickness), *n* (%)	31 (53.4)/21 (36.2)/6 (10.3)	24 (40.7)/28 (47.5)/7 (11.9)	χ^2^ = 1.09	0.579
High-tension area, *n* (%)	22 (37.9)	20 (33.9)	χ^2^ = 0.07	0.795
Edge regularity (Regular/Irregular), *n* (%)	41 (70.7)/17 (29.3)	40 (67.8)/19 (32.2)	χ^2^ = 0.21	0.651
Contamination grade (Clean/Contaminated/Severely contaminated), *n* (%)	34 (58.6)/20 (34.5)/4 (6.9)	37 (62.7)/16 (27.1)/6 (10.2)	χ^2^ = 0.77	0.680
Anesthesia type (Local/Regional block/General), *n* (%)	43 (74.1)/8 (13.8)/7 (12.1)	48 (81.4)/5 (8.5)/6 (10.2)	χ^2^ = 1.04	0.596
Debridement type (Irrigation only/Standard/Extensive), *n* (%)	15 (25.9)/37 (63.8)/6 (10.3)	14 (23.7)/33 (55.9)/12 (20.3)	χ^2^ = 2.25	0.324
Prophylactic antibiotics, *n* (%)	40 (69.0)	38 (64.4)	χ^2^ = 0.11	0.744
Layered closure, *n* (%)	30 (51.7)	36 (61.0)	χ^2^ = 0.68	0.408
Suture material [Nylon/Polypropylene/Absorbable (Vicryl)], *n* (%)	22 (37.9)/18 (31.0)/18 (31.0)	25 (42.4)/12 (20.3)/22 (37.3)	χ^2^ = 1.78	0.410

BMI, body mass index; DM, diabetes mellitus; SD, standard deviation; IQR, interquartile range.

### 3.2 Wound healing outcomes and wound-related complications

The median time to complete healing was 9.82 [8.38, 11.46] days in the aesthetic suturing group and 10.31 [9.02, 12.86] days in the conventional suturing group, without a statistically significant between-group difference (Mann–Whitney U = 1,498, *P* = 0.248). The primary healing rate was comparable between groups (81.0% vs. 81.4%; *χ*^2^ = 0.00, *P* = 0.965). The incidence of SSI was identical (8.6% vs. 8.5%; Fisher's exact test, *P* = 1.000). No statistically significant differences were observed for wound dehiscence (1.7% vs. 6.8%; Fisher's exact test, *P* = 0.364), hematoma/seroma (5.2% vs. 3.4%; Fisher's exact test, *P* = 0.679), necrosis/marginal ischemia (5.2% vs. 3.4%; Fisher's exact test, *P* = 0.679), or the requirement for secondary suturing/re-debridement (3.4% vs. 3.4%; Fisher's exact test, *P* = 1.000). Notably, revisit or readmission occurred less frequently in the aesthetic suturing group than in the conventional suturing group (1.7% vs. 18.6%; *χ*^2^ = 9.10, *P* = 0.003) (Table 2). The single unscheduled revisit/readmission in the aesthetic suturing group was related to wound concern/delayed healing. In the conventional suturing group, the primary reasons were wound concern/delayed healing (4/11, 36.4%), suspected infection (4/11, 36.4%), and dressing- or review-related needs (2/11, 18.2%); cosmetic dissatisfaction was less common (1/11, 9.1%).

**Table 2:** Wound healing outcomes and wound-related complications.

Outcome	Aesthetic suturing (*n* = 58)	Conventional suturing (*n* = 59)	Test statistic	*P* value
Time to complete healing, days	9.82 [8.38, 11.46]	10.31 [9.02, 12.86]	U = 1,498	0.248
Primary healing, *n* (%)	47 (81.0)	48 (81.4)	χ^2^ = 0.00	0.965
Surgical site infection (SSI), *n* (%)	5 (8.6)	5 (8.5)	Fisher's exact	1.000
Wound dehiscence, *n* (%)	1 (1.7)	4 (6.8)	Fisher's exact	0.364
Hematoma/seroma, *n* (%)	3 (5.2)	2 (3.4)	Fisher's exact	0.679
Necrosis/marginal ischemia, *n* (%)	3 (5.2)	2 (3.4)	Fisher's exact	0.679
Secondary suturing/re-debridement, *n* (%)	2 (3.4)	2 (3.4)	Fisher's exact	1.000
Revisit or readmission, *n* (%)	1 (1.7)	11 (18.6)	χ^2^ = 9.10	0.003

SSI, surgical site infection; IQR, interquartile range.

### 3.3 Scar formation and aesthetic outcomes

The aesthetic suturing group demonstrated significantly lower VSS total scores compared with the conventional suturing group [1.00 (0.00, 4.75) vs. 7.00 (2.50, 12.00); Mann–Whitney U = 812, *P* < 0.001]. Consistently, all VSS subdomains favored aesthetic suturing, including vascularity [0.00 (0.00, 1.00) vs. 2.00 (1.00, 3.00); U = 812, *P* < 0.001], pigmentation [0.00 (0.00, 1.00) vs. 1.00 (0.00, 2.00); U = 914, *P* < 0.001], pliability [1.00 (0.00, 2.00) vs. 2.00 (1.00, 5.00); U = 916, *P* < 0.001], and height/thickness [0.00 (0.00, 1.00) vs. 2.00 (0.00, 2.50); U = 949, *P* < 0.001]. Adverse scar events did not differ significantly between groups. Hypertrophic scar formation occurred in 13.8% of patients in the aesthetic suturing group and 25.4% in the conventional suturing group (*χ*^2^ = 2.51, *P* = 0.113), while scar contracture was observed in 6.9% and 6.8%, respectively (Fisher's exact test, *P* = 1.000). Regarding scar-related symptoms, scar pain scores were lower in the aesthetic suturing group [1.48 (0.61, 1.90) vs. 1.99 (1.10, 2.81); U = 1,282, *P* = 0.019], whereas itch scores did not differ significantly [1.47 (0.71, 2.33) vs. 2.08 (1.21, 2.96); U = 1,430, *P* = 0.127]. Patient-reported satisfaction was higher in the aesthetic suturing group [3.00 (3.00, 4.00) vs. 2.00 (1.00, 3.00); U = 2,768, *P* < 0.001] (Table 3). Scar assessments used for the primary analysis were based on the follow-up visit closest to 3 months after wound closure. The median follow-up duration for scar assessment was 3.10 [2.85, 3.35] months in the aesthetic suturing group and 3.09 [2.80, 3.38] months in the conventional suturing group (Mann–Whitney U = 1,692, *P* = 0.824).

**Table 3:** Scar-related outcomes and patient-reported aesthetic satisfaction.

Outcome	Aesthetic suturing (*n* = 58)	Conventional suturing (*n* = 59)	Test statistic	*P* value
VSS total score	1.00 [0.00, 4.75]	7.00 [2.50, 12.00]	U = 812	<0.001
VSS vascularity	0.00 [0.00, 1.00]	2.00 [1.00, 3.00]	U = 812	<0.001
VSS pigmentation	0.00 [0.00, 1.00]	1.00 [0.00, 2.00]	U = 914	<0.001
VSS pliability	1.00 [0.00, 2.00]	2.00 [1.00, 5.00]	U = 916	<0.001
VSS height/thickness	0.00 [0.00, 1.00]	2.00 [0.00, 2.50]	U = 949	<0.001
Hypertrophic scar, *n* (%)	8 (13.8)	15 (25.4)	χ^2^ = 2.51	0.113
Scar contracture, *n* (%)	4 (6.9)	4 (6.8)	Fisher's exact	1.000
Scar itch (NRS), points	1.47 [0.71, 2.33]	2.08 [1.21, 2.96]	U = 1,430	0.127
Scar pain (NRS), points	1.48 [0.61, 1.90]	1.99 [1.10, 2.81]	U = 1,282	0.019
Patient satisfaction (Likert 1–5)	3.00 [3.00, 4.00]	2.00 [1.00, 3.00]	U = 2,768	<0.001

VSS, Vancouver scar scale; NRS, numeric rating scale; IQR, interquartile range.

### 3.4 Subgroup analyses and tests for interaction

Subgroup analyses of VSS total score are presented in Table 4. In the face subgroup, aesthetic suturing was associated with a lower VSS total score compared with conventional suturing [0.00 (0.00, 2.00) vs. 6.50 (2.00, 9.00); U = 654, *P* < 0.001]; a similar direction and magnitude of effect was observed in the non-face subgroup [2.00 (0.00, 6.00) vs. 8.00 (3.00, 13.00); U = 686, *P* < 0.001], with no evidence of interaction by wound location (P_interaction=0.601). The association remained directionally consistent in high-tension and low-tension strata (both *P* < 0.001), and interaction testing did not indicate effect modification (P_interaction=0.632). When stratified by contamination severity, the effect was evident in the clean/mild contamination stratum (*P* < 0.001), whereas estimates were imprecise in the marked contamination stratum due to limited sample size (*P* = 0.286); the interaction term was not statistically significant (P_interaction=0.610). Stratification by time-to-suturing proxy (≤8 h vs. >8 h) and by age (<65 vs. ≥65 years) showed no statistically significant interaction (P_interaction=0.545 and 0.847, respectively). Overall, interaction testing suggested that the association between aesthetic suturing and lower VSS total score was broadly consistent across prespecified subgroups, while small stratum sizes (notably marked contamination and >8 h) limited precision.

**Table 4:** Subgroup analyses for VSS total score and interaction testing.

Subgroup (VSS_total endpoint)	Aesthetic *n*/Total	Aesthetic VSS_total	Conventional VSS_total	Median difference	Within-subgroup test	*P* value
Wound location (P_interaction=0.601)						
Face	26/44	0.00 [0.00, 2.00]	6.50 [2.00, 9.00]	−6.50	U = 654	<0.001
Non-face	32/73	2.00 [0.00, 6.00]	8.00 [3.00, 13.00]	−6.00	U = 686	<0.001
Tension/joint area (proxy) (P_interaction=0.632)						
High-tension area	22/42	2.00 [0.00, 6.00]	8.00 [4.00, 13.00]	−6.00	U = 300	<0.001
Low-tension area	36/75	1.00 [0.00, 4.00]	6.00 [2.00, 10.00]	−5.00	U = 742	<0.001
Contamination (proxy) (P_interaction=0.610)						
Clean/mild contamination	54/107	1.00 [0.00, 4.00]	7.00 [2.00, 12.00]	−6.00	U = 759	<0.001
Marked contamination	4/10	6.50 [1.00, 13.25]	10.50 [6.00, 14.00]	−4.00	U = 4	0.286
Time from injury to suturing (proxy) (P_interaction=0.545)						
≤8 h	57/114	1.00 [0.00, 4.00]	7.00 [2.50, 12.00]	−6.00	U = 792	<0.001
>8 h	1/3	1.00 [1.00, 1.00]	6.00 [6.00, 6.00]	−5.00	U = 0	0.333
Age (P_interaction=0.847)						
Non-elderly (<65 years)	56/112	1.00 [0.00, 4.00]	7.00 [2.00, 12.00]	−6.00	U = 775	<0.001
Elderly (≥65 years)	2/5	2.50 [0.50, 4.50]	7.00 [7.00, 7.00]	−4.50	U = 1	1.000

VSS, Vancouver scar scale; IQR, interquartile range.

## 4 Discussion

In this retrospective cohort of patients with traffic accident–related injuries requiring primary wound closure, aesthetic suturing was associated with significantly improved scar quality and higher patient-reported satisfaction compared with conventional suturing, while early wound healing kinetics and most wound-related complications were comparable between groups. Notably, time to complete healing, primary healing rate, and the incidence of surgical site infection, dehiscence, hematoma/seroma, and marginal ischemia did not differ significantly, suggesting that the adoption of cosmetic-oriented closure principles did not compromise short-term wound safety in this trauma setting. The most prominent benefit of aesthetic suturing was observed in downstream scar outcomes, including lower Vancouver Scar Scale scores across all domains and reduced scar-related pain, accompanied by fewer unscheduled revisits or readmissions. These findings highlight that, in traumatic lacerations amenable to direct closure, technical refinements aimed at tension redistribution, precise dermal alignment, and atraumatic handling may meaningfully influence scar maturation and patient perception without altering early epithelial repair. From a clinical perspective, this supports the integration of aesthetic suturing principles into routine trauma wound management, particularly for wounds located in cosmetically sensitive or functionally mobile areas, where long-term scar appearance and patient satisfaction are important determinants of overall treatment success (15, 16).

The absence of a statistically significant between-group difference in time to complete healing (median 9.82 vs. 10.31 days) suggests that the epithelialization phase and early wound closure were not substantially altered by the suturing approach in this setting. This is consistent with the concept that, for cleanly approximated traumatic lacerations that receive adequate debridement and infection prophylaxis when indicated, the dominant drivers of re-epithelialization are perfusion adequacy, tissue viability, and control of bioburden rather than the specific aesthetic features of cutaneous suture placement. Contemporary syntheses of emergency department traumatic wound closure techniques similarly emphasize that cosmetic/functional endpoints often vary more than infection endpoints across closure approaches when core principles (irrigation, debridement, appropriate closure timing, and tension management) are met (7, 17).

With respect to wound-related complications, the comparable primary healing rate and the similar incidence of surgical site infection (SSI), dehiscence, hematoma/seroma, and marginal ischemia are clinically relevant. These findings indicate that aesthetic suturing did not increase early risk—an important consideration in traumatic wounds where contamination, tissue crush, and irregular edges may be present. Recent guidance and expert consensus for acute wound management highlight that infection prevention is primarily determined by wound cleansing, removal of devitalized tissue, and appropriate closure strategy (including delayed primary closure for selected high-risk wounds), rather than by refinements in epidermal suturing alone (18). An exception in the current dataset was the lower frequency of revisit/readmission in the aesthetic suturing group. Although this endpoint may reflect differences in clinical course, it can also be influenced by patient perception of scar/wound appearance, reassurance, discharge education, follow-up accessibility, and clinician thresholds for advising return. Therefore, while this finding is potentially meaningful from a health-service utilization perspective, residual confounding and measurement heterogeneity should be considered when attributing causality.

The most consistent signal in this study was the substantially lower VSS total score and favorable shifts across VSS subdomains (vascularity, pigmentation, pliability, and height/thickness) in the aesthetic suturing group. The observed improvement in scar outcomes after aesthetic suturing is biologically plausible. Cosmetic-oriented closure techniques emphasize meticulous wound-edge alignment, reduction of superficial and deep tension through layered approximation when indicated, and avoidance of excessive tissue strangulation (10). These principles may help preserve microvascular perfusion at the wound margins, reduce local inflammatory burden, and promote more organized collagen deposition during the remodeling phase. In traumatic lacerations, where edge irregularity and heterogeneous tension are common, such technical considerations may be particularly relevant to subsequent scar maturation. Scar quality is sensitive to the mechanical microenvironment during the proliferative and remodeling phases. Local tension influences inflammatory duration, fibroblast-to-myofibroblast differentiation, collagen deposition, angiogenesis, and extracellular matrix organization (19). Prior reviews on biomechanical forces in pathological scarring have shown that tension and tissue stiffness can amplify pro-fibrotic signaling and sustain matrix remodeling, supporting tension reduction as a practical anti-scar strategy (10, 20). Aesthetic suturing typically incorporates precise dermal alignment, layered closure for load distribution, and cutaneous techniques that minimize epidermal edge strangulation and suture-track inflammation. These elements reduce focal stress concentration and microischemia at the wound edge, which may translate into improved vascularity, pliability, and scar thickness during maturation. Experimental and translational studies suggest that mechanotransduction pathways may contribute to fibrotic vs. regenerative healing trajectories, further supporting the biological plausibility of tension modulation in scar prevention (21).

Scar pain was lower in the aesthetic suturing group, while itch did not differ statistically. Symptom trajectories after traumatic laceration repair are multifactorial and may reflect nerve fiber injury, local inflammation, and scar thickness/tension. The observed pattern—pain reduction without a clear itch difference—may indicate that aesthetic suturing influences mechanical irritation and local inflammation at the scar–skin interface without necessarily altering pruritus pathways, which can be sensitive to individual predisposition and environmental triggers. In parallel, patient satisfaction was higher in the aesthetic suturing group, which is concordant with lower VSS scores and aligns with clinical emphasis on patient-centered outcomes for visible or socially salient sites. Interpretation of VSS-based endpoints warrants attention to measurement properties. While VSS remains widely used and provides domain-level granularity, recent reviews highlight broader challenges across subjective scar assessment instruments, including inter-rater variability and ceiling/floor effects depending on scar type and follow-up duration (22, 23). Consequently, triangulation with additional outcomes would strengthen inference in future work.

Across prespecified subgroups, aesthetic suturing remained associated with lower VSS total scores, and interaction testing did not indicate statistically significant effect modification by wound location (face vs. non-face), tension zone, contamination category, time to suturing proxy, or age category. Clinically, this suggests that the cosmetic advantage was not restricted to a single anatomic region and may generalize across typical traumatic laceration distributions, though the precision in smaller strata (notably marked contamination and >8-hour closure) was limited. This limitation is consistent with general challenges in subgroup inference: when stratum sizes are small, confidence intervals widen and interaction tests are underpowered even when directionality is consistent.

The current findings are broadly aligned with contemporary evidence that closure technique can meaningfully influence cosmetic outcomes without necessarily changing infection rates when wounds are appropriately selected and managed. A recent systematic review focusing on traumatic wound closure in emergency settings describes heterogeneity across techniques for cosmetic outcomes and emphasizes that infection-related outcomes often depend more on wound and process factors than on fine technical variations in cutaneous suturing (7). Randomized comparisons in laceration repair contexts also reinforce that cosmetic outcomes can vary across closure modalities when assessed by caregivers or patients, supporting the relevance of technique selection to perceived appearance (24, 25). Although such studies differ in patient population and wound characteristics from traffic accident–related injuries, they provide convergent evidence that cosmetic endpoints are sensitive to closure approach even when short-term wound complication rates remain similar.

Several limitations should be considered. First, the retrospective observational design is inherently susceptible to selection bias and residual confounding. Clinician choice of suturing technique may have been influenced by unmeasured factors such as wound complexity, patient preference, or anticipated cosmetic priority. In addition, wound length and area were greater in the conventional suturing group at baseline. Although stratified analyses demonstrated directionally consistent associations across wound size–related subgroups, residual confounding related to baseline wound dimensions cannot be fully excluded. Second, follow-up completeness and timing may have influenced scar-related outcomes. Scar maturation evolves over months, and variability in assessment windows may affect Vancouver Scar Scale values. Moreover, VSS is a semi-quantitative and observer-dependent instrument; in the absence of blinded evaluators and formal inter-rater reliability testing, differential assessment bias cannot be ruled out. Third, certain exploratory subgroups, particularly the marked contamination, delayed closure beyond 8 h, and elderly strata, were small in size. Although the overall direction of effect was consistent, limited sample sizes reduce statistical precision and constrain the power to detect interaction effects. Findings within these strata should therefore be interpreted cautiously. Fourth, the endpoint of revisit or readmission is multifactorial and may reflect nonclinical determinants such as access to care, discharge counseling, and patient anxiety, in addition to wound status, requiring cautious interpretation. Finally, the mechanistic interpretation offered in this study is based on existing experimental and translational literature rather than direct mechanistic measurements within our cohort. We did not assess biomechanical forces, tissue perfusion, inflammatory biomarkers, or molecular signaling pathways. Future prospective studies incorporating standardized scar assessment, longer follow-up, objective biomechanical or histologic evaluation, and adequately powered subgroup analyses are warranted to further clarify causal pathways and strengthen the generalizability of these findings.

## 5 Conclusion

Aesthetic suturing was associated with superior scar-related outcomes in patients with traffic accident–related injuries, evidenced by markedly lower VSS total and domain scores, reduced scar pain, and higher patient satisfaction. Early wound healing endpoints, including time to complete healing, primary healing, and most wound-related complications, were comparable between groups, while revisit/readmission was less frequent after aesthetic suturing. Subgroup analyses suggested consistent aesthetic benefit.

## Data Availability

The raw data supporting the conclusions of this article will be made available by the authors, without undue reservation.
